# Publisher Correction: RNase III-CLASH of multi-drug resistant *Staphylococcus aureus* reveals a regulatory mRNA 3′UTR required for intermediate vancomycin resistance

**DOI:** 10.1038/s41467-022-33167-2

**Published:** 2022-09-27

**Authors:** Daniel G. Mediati, Julia L. Wong, Wei Gao, Stuart McKellar, Chi Nam Ignatius Pang, Sylvania Wu, Winton Wu, Brandon Sy, Ian R. Monk, Joanna M. Biazik, Marc R. Wilkins, Benjamin P. Howden, Timothy P. Stinear, Sander Granneman, Jai J. Tree

**Affiliations:** 1 School of Biotechnology and Biomolecular Sciences, University of New South Wales, Sydney, NSW Australia; 2 Department of Microbiology and Immunology, Peter Doherty Institute, University of Melbourne, Melbourne, VIC Australia; 3 Centre for Systems and Synthetic Biology, University of Edinburgh, Edinburgh, UK; 4 Electron Microscopy Unit, University of New South Wales, Kensington, NSW Australia

**Keywords:** Bacterial genetics, Gene regulation, Antimicrobial resistance

Correction to: *Nature Communications* 10.1038/s41467-022-31177-8, published online 22 June 2022

The original version of this Article incorrectly cited itself in Ref. 32. The correct Ref. 32 is: “McKellar, S.W., et al. RNase III CLASH in MRSA uncovers sRNA regulatory networks coupling metabolism to toxin expression. *Nat. Commun*. **13**, 3560 (2022).”. This has now been corrected in both the PDF and HTML versions of the Article.

