# Supplementary material for: RNase III-CLASH of multi-drug resistant Staphylococcus aureus reveals a regulatory mRNA 3′UTR required for intermediate vancomycin resistance
Source: Nat Commun. 2022 Jun 22;13:3558. doi: 10.1038/s41467-022-31177-8 (PMC9217812; doi:10.1038/s41467-022-31177-8)
Supplement: Supplementary file 1 — Supplementary Information [file 41467_2022_31177_MOESM1_ESM.pdf]

RNase III-CLASH of multi-drug resistant *Staphylococcus aureus* reveals a regulatory mRNA 3'UTR required for intermediate vancomycin resistance

Daniel G. Mediati, Julia L. Wong, Wei Gao, Stuart McKellar, Chi Nam Ignatius Pang, Sylvania Wu, Winton Wu, Brandon Sy, Ian R. Monk, Joanna M. Biazik, Marc R. Wilkins, Benjamin P. Howden, Timothy P. Stinear, Sander Granneman, Jai J. Tree

SUPPLEMENTARY INFORMATION

Table of contents

Supplementary Figure 1……2
Supplementary Figure 2……3
Supplementary Figure 3……4
Supplementary Figure 4……6
Supplementary Figure 5……8
Supplementary Figure 6……9
Supplementary Figure 7……10
Supplementary Methods……11
Supplementary References……17

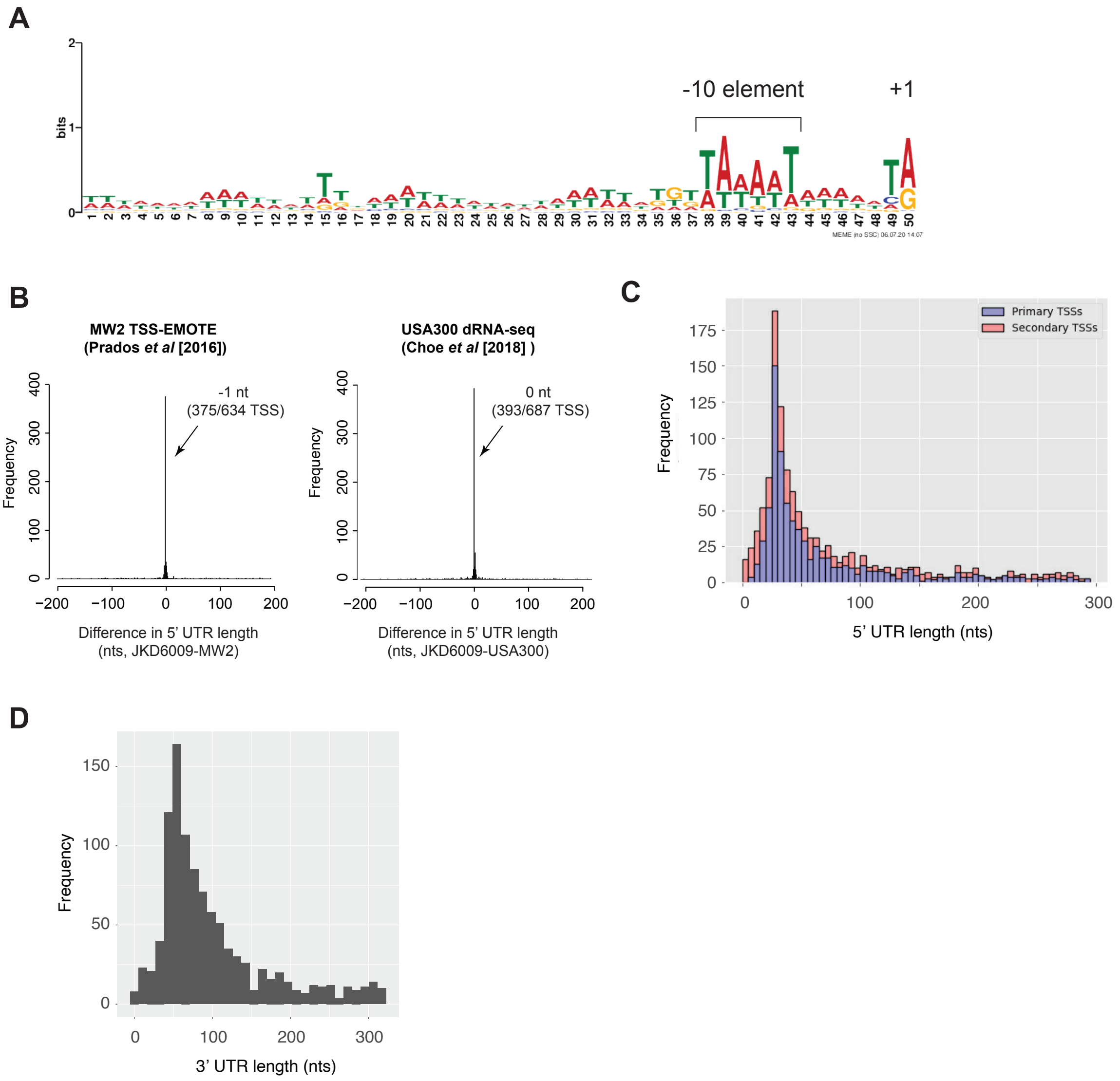

Supplementary Figure 1. Confirmation of RNA-end mapping data from *S. aureus* JKD6009. **A**. Motif analysis of the sequence upstream of primary transcription start sites identified by dRNA-seq and analysed by ANNOgesic. The RNA polymerase -10 element is indicates above the sequence logo. **B**. Comparison of dRNA-seq data from JKD6009 to TSS-EMOTE data from MW2 (left) and dRNA-seq data from USA300 (right). The 5'UTR lengths (primary transcription start sites) were compared for orthologues and the difference in 5' UTR length is shown on the x-axis. **C**. The distribution of JKD6009 5'UTR lengths for primary and secondary transcription start sites is shown. **D**. The distribution of JKD6009 3'UTR lengths is shown.

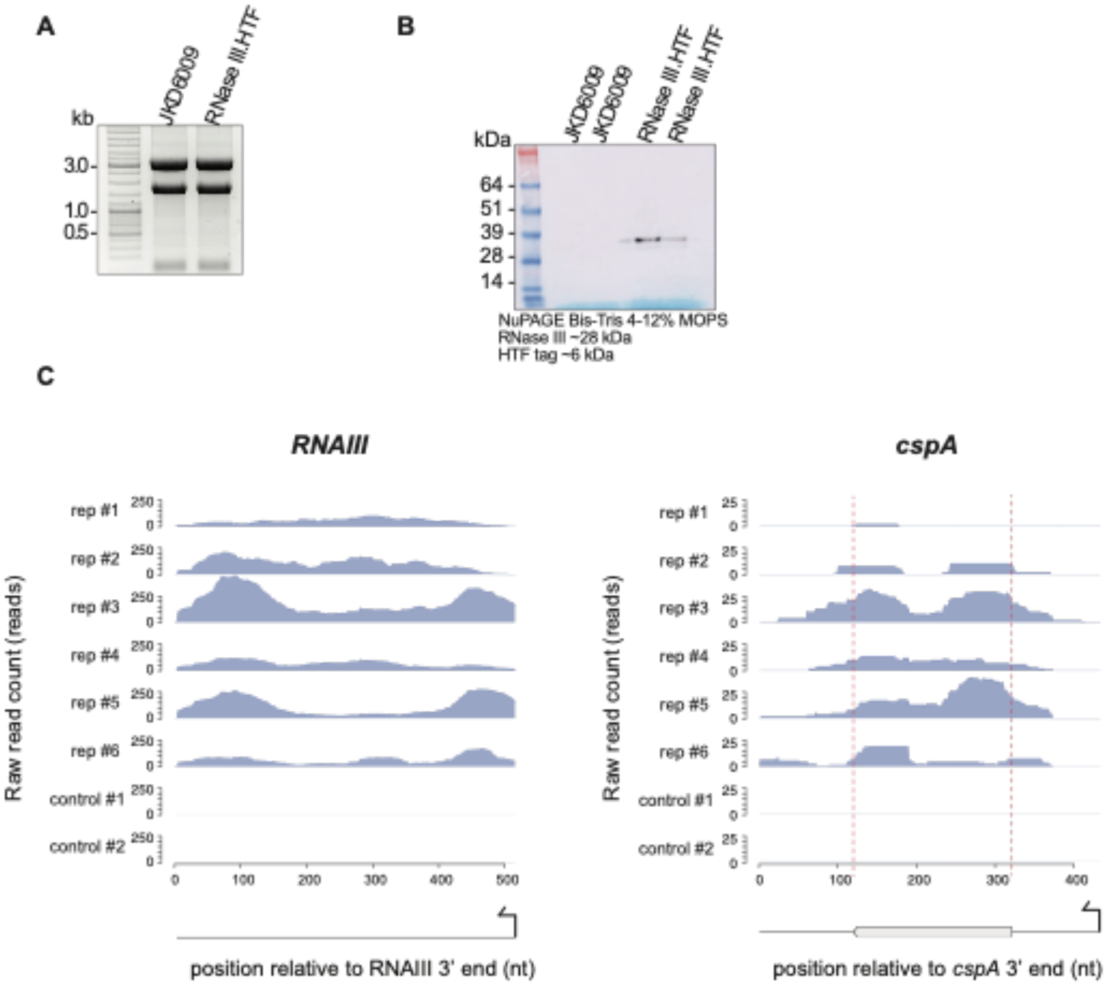

Supplementary Figure 2. Dual affinity tagging allows purification of RNase III and RNA targets. **A.** Electrophoresis of total RNA isolated from JKD6009 and the isogenic His-TEV-FLAG tagged RNase III strain indicates that both 23S and 16S rRNA have similar maturation. **B**. Anti-His Western blot of IMAC eluates from the untagged parental strain JKD6009 and isogenic *rnc-HTF* mutant. **C**. Reads recovered from RNase III CRAC map to the known RNase III targets RNAIII (left) and *cspA* (right). The raw read depth is shown relative to the 3' end of RNAIII and *cspA*.

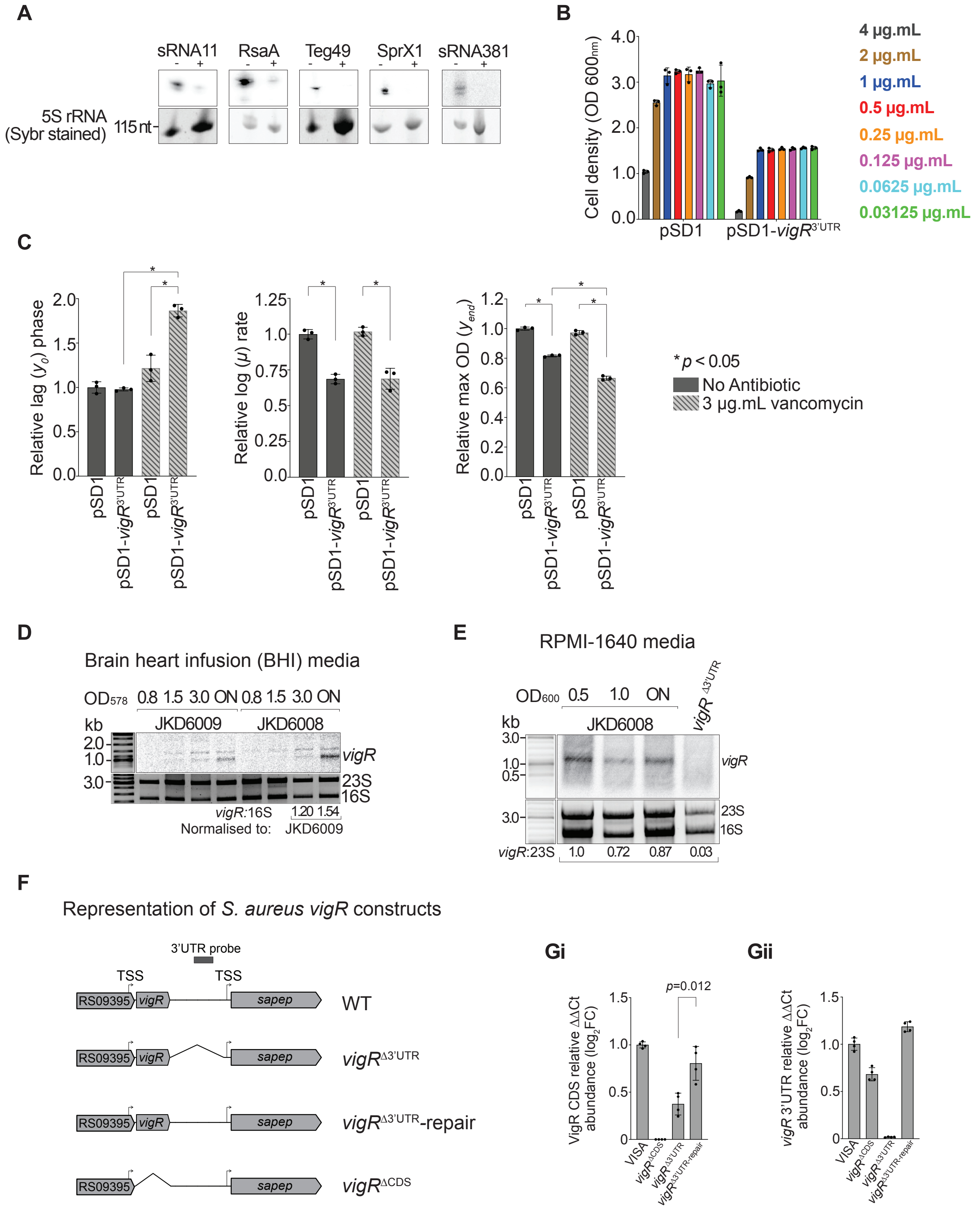

Supplementary Figure 3. VigR is required for glycopeptide tolerance in vancomycin-sensitive and vancomycin-intermediate *S. aureus*. **A.** Northern blot confirmation of CRISPRi knockdown strains. For each panel (target sRNA indicated above), total RNA was probed by Northern blot for the sRNA with the control vector pSD-1 (-) or with a sgRNA targeting the sRNA (+). 5S rRNA was used as a loading control (below). **B**. Broth microdilution assay of *S. aureus* JKD6008 with the CRISPRi control vector (pSD-1) and sgRNA targeting the *vigR* 3'UTR (pSD-1-*vigR*$^{3'UTR}$). Histogram heights represent the mean cell density (OD600$_{nm}$) and error bars indicate the standard deviation from *n*=3 biological replicates. **C.** Growth kinetics of *S. aureus* JKD6008 in the presence or absence of sub-inhibitory vancomycin with the

CRISPRi control vector (pSD-1) and sgRNA targeting the *vigR* 3'UTR (pSD-1-*vigR*$^{3'UTR}$). Plots display lag phase (y_0), log phase growth rate (μ), and maximal OD (y_{max}). Error bars indicate the standard deviation from n=3 biological replicates. Significance was calculated using a two-sided t-test. *p<0.05. **D&E**.Northern blot analysis of *vigR* mRNA expression during growth in BHI (**D**) and RPMI-1640 media (**E**). Total RNA from the vancomycin-sensitive strain JKD6009 or vancomycin-tolerant strain JKD6008 were probed at the growth stages indicated above. 5S rRNA was used a loading control. **F.** Schematic representation of *vigR* mutations constructed in this study. Bold line indicates the position of *vigR* 3'UTR northern probe. **G.** Quantification of (**i**) *vigR* CDS and (**ii**) *vigR*-3'UTR abundance in the CDS deletion, 3'UTR deletion, and 3'UTR-repaired backgrounds. qRT-PCR was used to quantify transcript and normalised to *gapA* abundance. Error bars represent the standard deviation from n=4 biological replicates. Significance was calculated using a two-sided t-test.

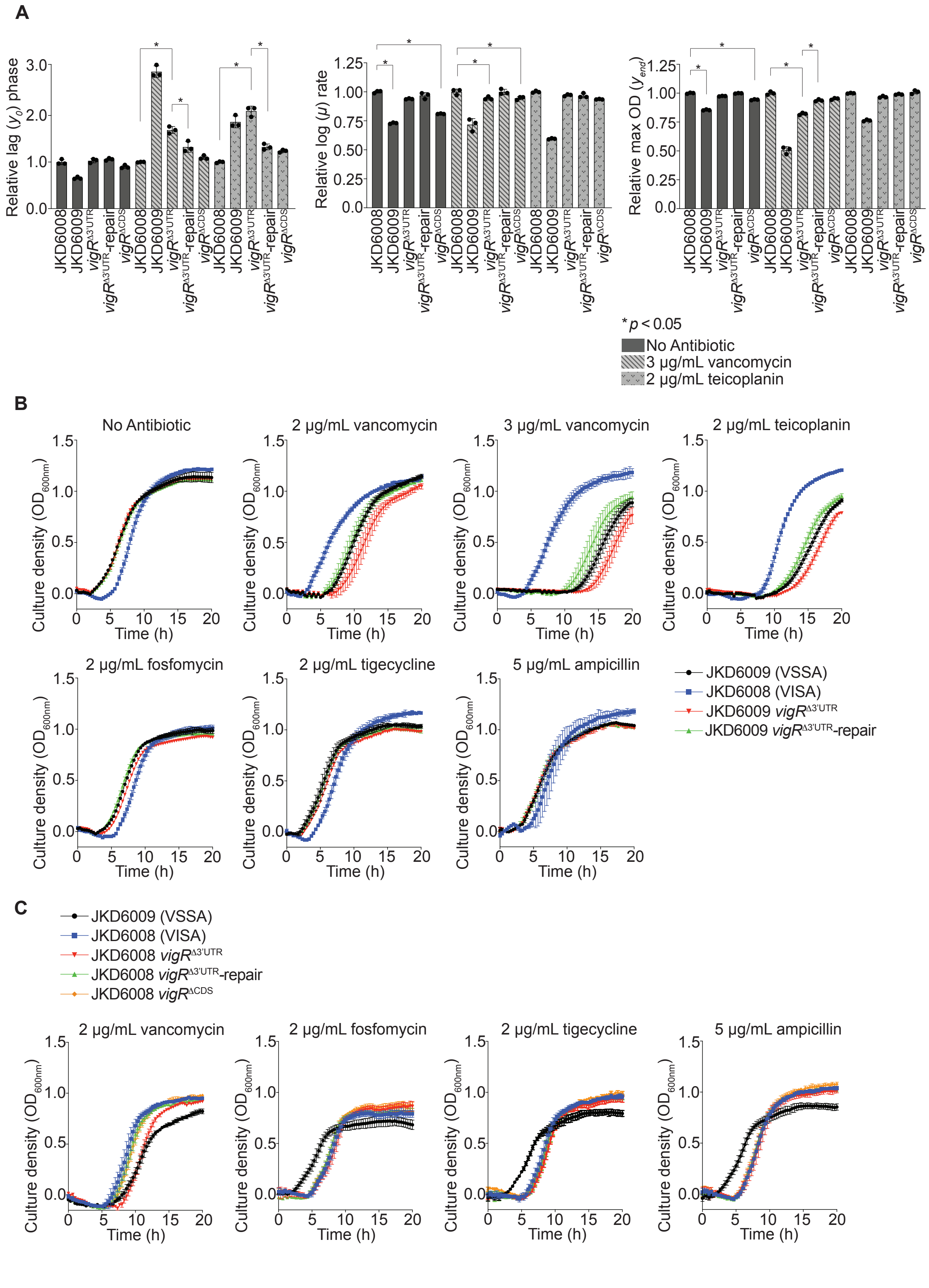

Supplementary Figure 4. Antibiotic sensitivity testing of the *vigR* 3'UTR deletion. **A**. Growth kinetics of JKD6008 (VISA), JKD6009 (VSSA), and isogenic JKD6008 deletions in the *vigR* CDS, 3'UTR, or 3'UTR-repaired strain in the presence of antibiotics (indicated). Plots display lag phase (y_0), log phase growth rate (μ), and maximal OD (y_{max}). Error bars indicate the standard deviation from n=3 biological replicates. Significance was calculated

using a two-sided *t*-test. **p*<0.05. **B**. Growth of the vancomycin-intermediate strain JKD6008 and isogenic mutants (indicated top) was monitored in Muller-Hinton media with the addition of antibiotics (indicated above plots). **C.** Growth of the vancomycin-sensitive strain JKD6009 and isogenic mutants (indicated top) was monitored in Muller-Hinton media with the addition of antibiotics (indicated above plots).

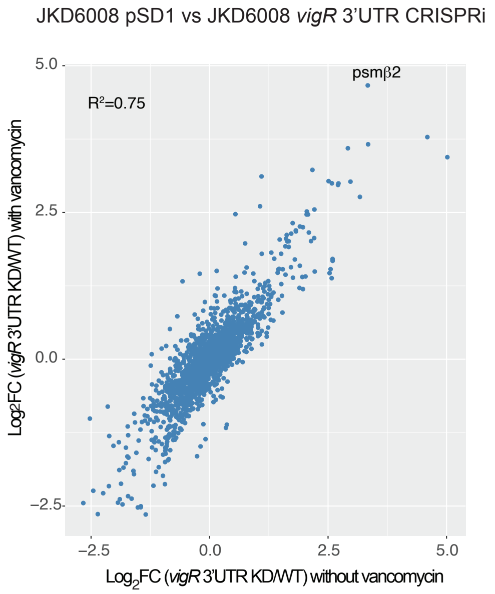

Supplementary Figure 5. Comparison of differential expression from the *vigR*-3'UTR knockdown strain with or without sub-inhibitory vancomycin (2µg/ml for 10 minutes).

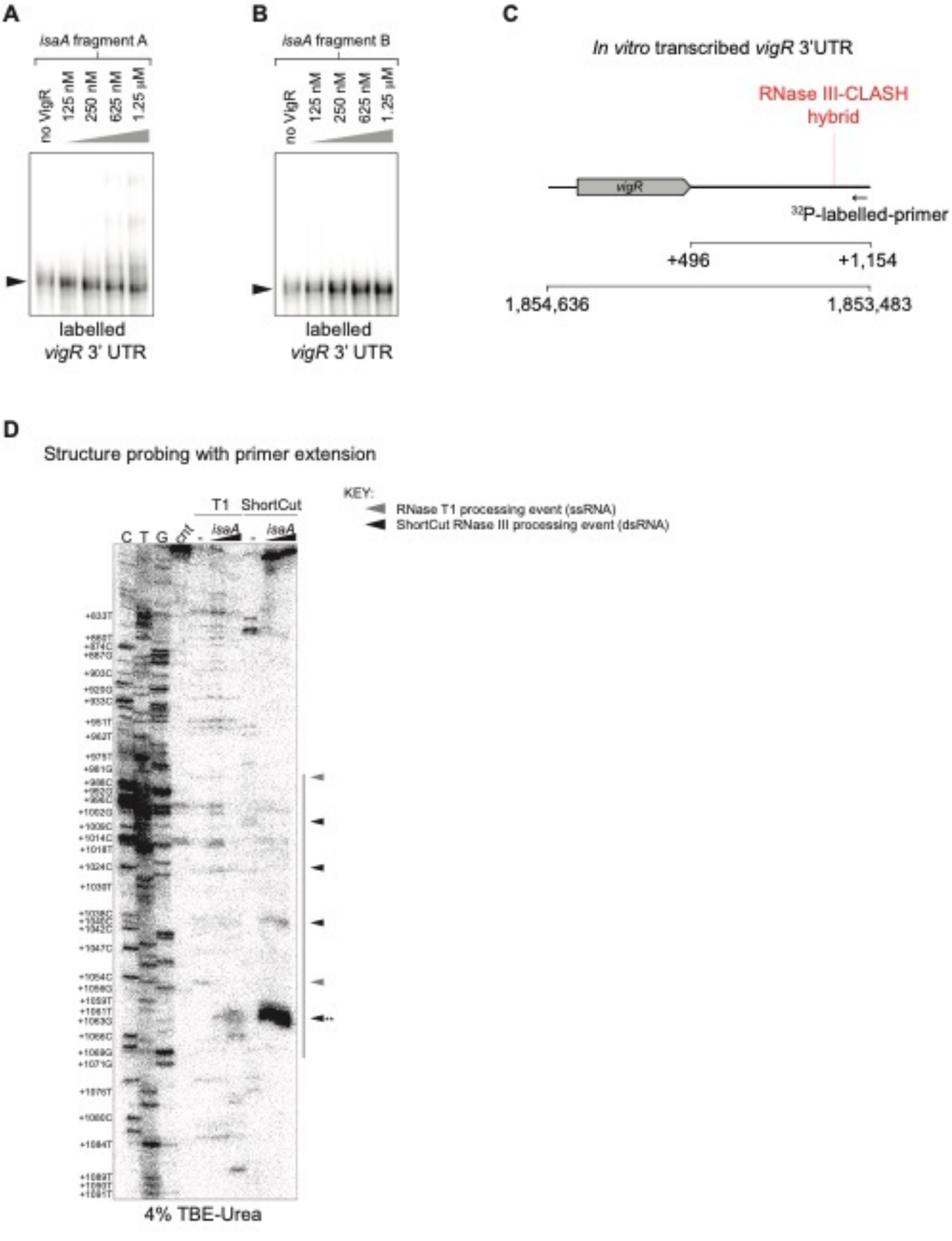

Supplementary Figure 6. The *isaA* mRNA and *vigR*-3'UTR interact *in vitro*. A. EMSA analysis of interaction between *vigR*-3'UTR and *isaA* mRNA fragment A (**A**) and fragment B (**B**). EMSA interactions with fragment C and shown in **Figure 6F & H**. **C.** Schematic representation of primer position for structure probing in D. **D.** Structure probing of the *vigR*-3'UTR interaction with i*saA* mRNA fragment C. Increase amounts of *isaA* fragment C was added to *vigR*-3'UTR and probed with RNase T1 or ShortCut RNase III.

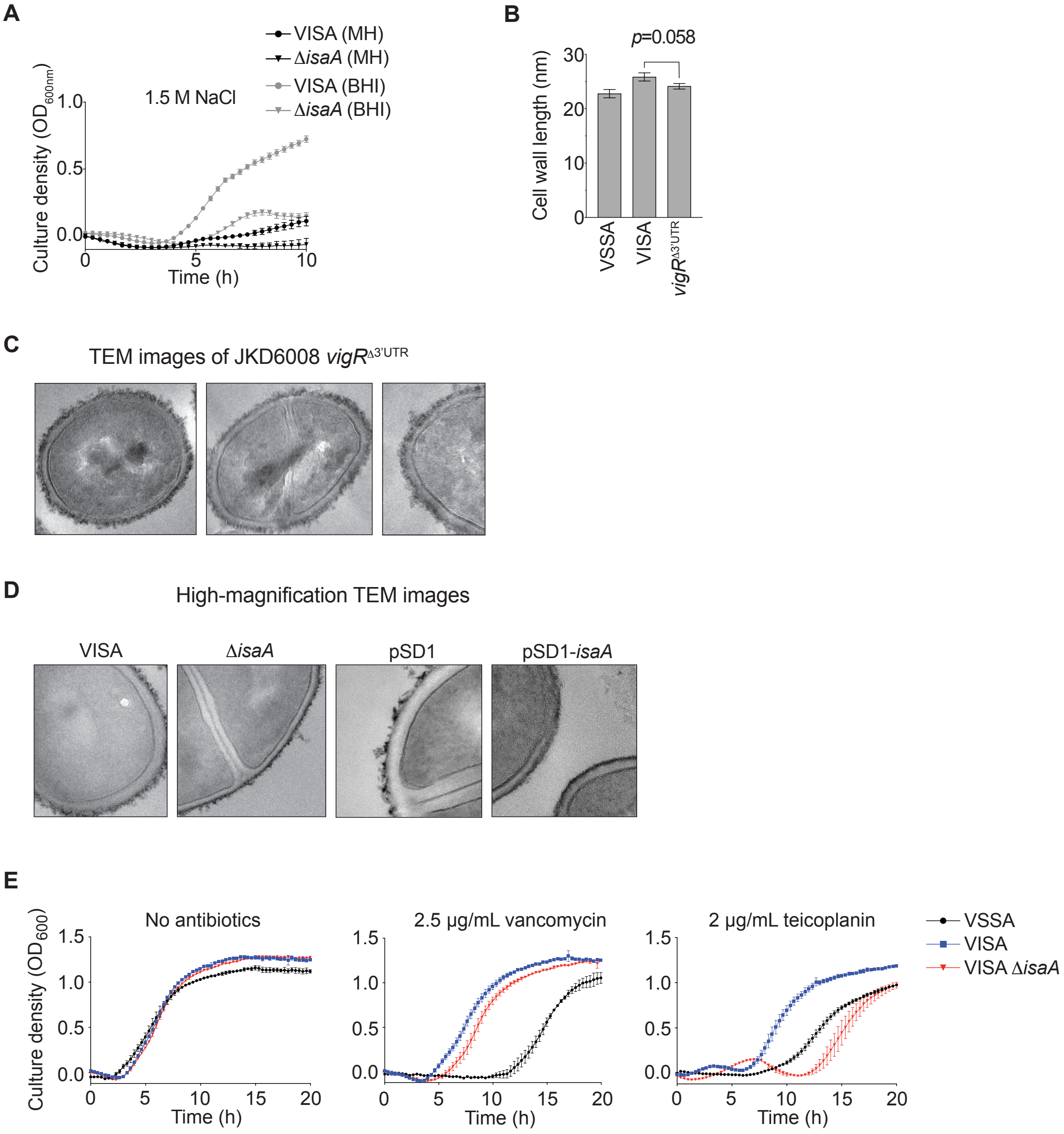

Supplementary Figure 7. The lytic transglycosylase IsaA is required for vancomycin tolerance. **A.** Analysis of JKD6008 and the isogenic *isaA* mutant growing in presence 1.5M NaCl added to either BHI or MH media. **B.** Quantification of cell wall thickness in JKD6009 (VSSA), JKD6008 (VISA), and JKD6008 *vigR* 3'UTR deletion strains. Histogram height represents mean and error bars indicate the standard error (SEM) from n=100 measurements. Significance was calculated using a two-sided t-test. p=0.058. **C.** High magnification transmission electron microscopy images of the JKD6008 *vigR* 3'UTR deletion. **D.** High magnification transmission electron microscopy images of JKD6008 and the isogenic deletion, and JKD6008 expressing the CRISPRi control vector (pSD1) and *isaA* knockdown strain (pSD1-*isaA*). **E.** Glycopeptide sensitivity testing of the JKD6008 *isaA* deletion. Growth of JKD6009, JKD6008, and JKD6008 *isaA* deletion (indicated top) was monitored in Muller-Hinton media with the addition of antibiotics (indicated above plots).

SUPPLEMENTARY METHODS

Analysis of Term-seq data. The FASTQ files generated were aligned to the *S. aureus* JKD6009 genome (NCBI: GCF_900607245.1) using the BWA 'mem' algorithm (Li and Durbin, 2009). Samtools (Li et al., 2009) was used to filter the aligned reads in BAM files format with the following parameters. Unmapped reads were excluded using the '-F 4' parameter, reads from secondary mapping were excluded using the '-F 256' parameter, and reads with MAPQ quality score less than 1 were excluded using the '-q 1' parameter. Multimapped reads were excluded from the BAM files by excluding rows of data with the following strings 'XA:Z:' and 'SA:Z:', which are strings that represent multimapped reads. To identify the genomic coordinate of the 3'-end of each read, the 'bedtools' (Quinlan and Hall, 2010) command line tool was utilized with the 'genomecov -d -5' option to analyse the 5'-end and with the '-strand +' or '-strand -' options to independently analyze each strand. Since the Term-seq protocol includes a PCR amplification step that reverses the strandedness of the sequence, to ensure the resulting data is consistent with the strandedness of the genes in the genome, the negative and positive strand results were swapped after the bedtools analysis above. The above steps result in one data file in graph (gr) file format for each input FASTQ file. Each graph file records each position along a single strand of the genome, along with the number of reads with transcript termination sites for each genomic position along the single strand. Normalisation was performed for each sample by dividing the read counts at each genomic position by the total number of termination sites in each sample and multiplying by a factor of 1 x 10^6. The above normalization step results in a counts per million (cpm) value for the termination sites at each genomic position.

To identify the peaks that represent the transcript termination sites, the 'peakPick' R library (Weber et al., 2014) was adapted for this purpose. In brief, the 'peakPick' algorithm uses a sliding window algorithm to detect peaks. Each window is defined as the region that includes a central position in the genome and the region plus or minus a user defined number of nucleotides around the central position. The mean and standard error of the mean (SEM) for the number of reads with termination sites across each genomic position within the window was calculated. A peak termination site is detected at the central position of the window if the number reads with termination sites is above the local mean plus a user-defined multiple of the SEM. The algorithm is iterative in that previously detected peaks were excluded from the estimation of the mean and the SEM within the sliding window. The iteration stops when the number of new peaks identified is equal to or less than the last iteration. This results in a set of termination sites for each sample. The parameters that could be adjusted include the window length and the number of SEM above mean.

To fine-tune the analysis by the 'peakPick' software, a set of scripts were developed to fine-tune the choice of parameters and to summarise groups of peaks from replicate samples. Different parameters combination of window lengths and number of SEMs were tested to maximise the number of groups of peaks that were shared by multiple biological replicates. Since there are often peaks that are close to each other, peaks were merged into the same peak group if they are within 5 nucleotides of each other. The peak with the maximum height

within each peak group was selected as the representative transcript termination site. It was found that a window size of 8 and peak heights of 7x SEM above the peak maximises the number peak groups shared between biological replicates. The scripts from TermPick pipeline for the analysis of Term-seq data are available in the following GitHub repository (https://github.com/IgnatiusPang/TermPick).

ANNOgesic. The ANNOgesic pipeline (Yu et al., 2018) was used to integrate transcriptomics data generated from RNA-seq, dRNA-seq (Sharma and Vogel, 2014) and Term-seq (Dar et al., 2016) to provide a detailed annotation of the *S. aureus* JKD6009 genome. The transcription start sites were identified using the TSSPredator (Dugar et al., 2013) module within the ANNogesic pipeline using the following parameters "--height 1.0 --height_reduction 0.5 --factor 2 --factor_reduction 0.5 --base_height 0 --enrichment_factor 3 --processing_factor 1 --validate_gene --replicate_tex all_1". The transcription start sites and the processing sites was identified by running the TSSpredator module successively with the options "--program TSS" followed by "--program PS". The results from the analysis of the Term-Seq data with the TermPick software was included into the ANNOgesic pipeline, with the terminator region including 20 nucleotides upstream from the termination site, with a total of 21 nucleotides including the termination site. Additional analyses performed using the ANNogesic pipeline include the identification of sRNA, riboswitch, promoters and operons. The analyses resulted in a GFF file containing the detailed annotation of *S. aureus* JKD6009 genome. This GFF file was used in subsequent CLASH data analyses.

RNase III-CLASH, protocol A. JKD6009 WT and RNase III.HTF were used to inoculate 800 mL of pre-warmed liquid BHI and grown at 37°C to an OD_{578} 3.0 with 200 rpm shaking. Replicates 3-6 were crosslinked with 1800 mJ using a W5 small diameter UV-crosslinking unit (UV03, UK) and harvested by centrifugation (7000 *g* for 7 min). The supernatant was discarded, and cell pellets were resuspended in 50 mL cold PBS and centrifuged again (4000 *g* for 20 min). One volume of lysis buffer (50 mM Tris-HCl (pH 7.8), 150 mM NaCl, 5mM β-mercaptoethanol, 0.1% NP-40, 0.5% Triton X-100 and 1 tablet of "cOmplete" EDTA-free protease inhibitor (Roche) per 50 mL of buffer) and 100 μg/mL of Lysostaphin (Sigma) was added to each pellet and allowed to incubate on ice for 30 min. Three volumes of 0.1-mm zirconia beads were added to each pellet and vortexed for 5 x 1 min intervals with 1 min on ice between each interval. Cell debris was centrifuged (3,200 *g* for 20 min) and the clarified lysate was transferred to 1.5 mL microcentrifuge tubes and further clarified at 16,000 *g* for 20 min. Supernatants were added to equilibrated M2 anti-FLAG resin (Sigma) and incubated at 4°C with gentle rotation for 2 h. The resin was washed twice in 10 mL TNM1000 buffer (50 mM Tris-HCl (pH 7.8), 1 M NaCl, 5mM β-mercaptoethanol, 0.1% NP-40, 0.5% Triton X-100, 1 mM EDTA) and twice in 10 mL TNM150 (50 mM Tris-HCl (pH 7.8), 150 mM NaCl, 5mM β-mercaptoethanol, 0.1% NP-40, 0.5% Triton X-100). The protein-bound resin was resuspended in 300 μL of TNM150 and incubated with 50 U of GST.TEV protease at 18°C with gentle rotation for 2 h. Eluates were collected by filtration through a Bio-spin chromatography column (Bio-Rad) and incubated with 0.15 U of RNace-IT (Agilent) at 20°C for 5 min. The digestion stopped by the addition of 0.4 g guanidine-HCl, 300 mM NaCl and

10 mM imidazole (pH 8.0). Eluates were added to 100 μL Ni-NTA agarose resin (Thermo) equilibrated with wash buffer 1 (4 M guanidine-HCl, 50 mM Tris-HCl (pH 7.8), 300 mM NaCl, 5mM β-mercaptoethanol, 0.1% NP-40) and incubated at 4°C with gentle rotation for at least 18 h. Nickel agarose resin was washed once with 800 μL wash buffer 1 and three times with 800 μL PNK buffer (50 mM Tris-HCl (pH 7.8), 10 mM MgCl, 0.1% NP-40, 5mM β-mercaptoethanol). The resin was resuspended in 80 μL NP-PNK buffer (50 mM Tris-HCl (pH 7.8), 10 mM MgCl, 5mM β-mercaptoethanol) containing 8 U of thermosensitive alkaline phosphatase (TSAP, Promega) and 80 U of RNasin (Promega) and incubated at 20°C with rotation for 1 h. Ni-NTA resin was washed once with 800 μL wash buffer 1 and three times with 800 μL PNK buffer. The resin was resuspended in 80 μL NP-PNK buffer containing 20 U of T4 polynucleotide kinase (NEB), 20 U of RNasin (Promega) and 30 μCi ^{32}P-ATP and incubated at 20°C with rotation for 100 min. Cold 1 mM ATP was spiked in and reaction allowed to proceed for another 50 min. Ni-NTA resin was again washed twice with 800 μL wash buffer 1 and four times with 800 μL PNK buffer and then resuspended in 80 μL of NP-PNK buffer containing 40 U of T4 RNA ligase I (NEB), 1 mM ATP, 20 U of RNasin (Promega) and 100 pM of an L5 index barcoded 5' linker (Integrated DNA Technologies). The ligation reaction was incubated at 16°C with gentle rotation for at least 12 h. The Ni-NTA resin was washed twice with 800 μL wash buffer 1 and four times with 800 μL PNK buffer, and resuspended in 60 μL NP-PNK buffer containing 40 U of T4 RNA ligase I (NEB), 20 U of RNasin (Promega) and 80 pM of 3' linker App-PE (Integrated DNA Technologies). The ligation reaction was incubated at 16°C with gentle rotation for 6 h and then Ni-NTA resin was washed twice with 800 μL wash buffer 1 and four times with 800 μL wash buffer 2 (50 mM Tris-HCl (pH 7.8), 50 mM NaCl, 0.1% NP-40, 5mM β-mercaptoethanol, 10 mM imidazole). ^{32}P-labelled RNase III.RNA complexes were eluted in 60 μL of 1x NuPAGE LDS buffer (Thermo) and incubated at 65°C for 10 min with gentle shaking. Eluates were magnetically separated and loaded onto a NuPAGE 4-12% gradient Bis-Tris PAGE gel (Invitrogen) and run in 1x NuPAGE running buffer for ~1 h at 125 V. ^{32}P-labelled RNase III.RNA complexes were visualised by autoradiography, gel excised and recovered by incubating the fragmented gel pieces in 600 μL of wash buffer 3 (50 mM Tris-HCl (pH 7.8), 50 mM NaCl, 0.1% NP-40, 5mM β-mercaptoethanol, 1% SDS, 5 mM EDTA) and 100 μg of proteinase K at 55°C for 2 h with shaking. RNA was extracted using 3 M NaOAc and phenol:chloroform:isoamylalcohol, ethanol precipitated and pellet resuspended in 13 μL of RT buffer (RT_PE_reverse oligonucleotide, 5 mM dNTPs, H2O) and reverse-transcribed using Superscript III according to manufacturer's instructions. cDNA was incubated with RNase H (Thermo) at 37°C for 1 h and amplified using Phusion Hot Start Flex DNA Polymerase (NEB), 10 μM P5 and BC1 oligonucleotides. The cDNAs were amplified for 24 cycles with 2 μL of cDNA used as template. Products were separated on a 1.5% metaphor agarose gel and amplicons excised and purified using the MiniElute gel extraction kit (Qiagen). Libraries were pooled to equimolar, quantified on an Agilent bioanalyzer and sequenced on an Illumina NextSeq500 platform at Ramaciotti Centre for Genomics (University of New South Wales, Sydney), generating 150-cycle paired-end reads.

RNase III-CLASH, protocol B. JKD6009 WT and RNase III.HTF were used to inoculate 400 mL of pre-warmed liquid BHI and grown at 37°C to an OD_{578} 3.0 with 200 rpm shaking Replicates 1-2 were crosslinked with 400 mJ of UV irradiation using the Vari-X-linker (UVO3, UK) and immediately harvested by vacuum onto a membrane filter. Two mL of lysis buffer (50 mM Tris-HCl (pH 7.8), 150 mM NaCl, 5mM β-mercaptoethanol and 1 tablet of "cOmplete" EDTA-free protease inhibitor (Roche) per 50 mL of buffer) and 1 V of 0.1-mm zirconia beads were added to each cell pellet and vortexed for 1 min using the FastPrep-24 5G (MP Biomedicals). Dry ice was used to ensure cell pellets did not overheat. Cell debris was centrifuged (3,800 *g* for 10 min) and the clarified lysate was transferred to 1.5 mL microcentrifuge tubes and further clarified at 16,000 *g* for 20 min. Supernatants were added to equilibrated M2 anti-FLAG resin (Sigma) and incubated at 4°C with gentle rotation for 2 h. The resin was washed twice in 10 mL TNM1000 buffer (50 mM Tris-HCl (pH 7.8), 1 M NaCl, 5mM β-mercaptoethanol) and twice in 10 mL TNM150 (50 mM Tris-HCl (pH 7.8), 150 mM NaCl, 5mM β-mercaptoethanol, 0.1% NP-40). The protein-bound resin was resuspended in 300 µL of TNM150 and incubated with 50 U of GST.TEV protease at 18°C with gentle rotation for 2 h. Eluates were collected by filtration through a Bio-spin chromatography column (Bio-Rad) and incubated with 0.15 U of RNace-IT (Agilent) at 20°C for 5 min. The digestion stopped by the addition of 0.4 g guanidine-HCl, 300 mM NaCl and 10 mM imidazole (pH 8.0). Eluates were added to 100 µL magnetic Ni-NTA resin (Thermo) equilibrated with wash buffer 1 (4 M guanidine-HCl, 50 mM Tris-HCl (pH 7.8), 300 mM NaCl, 5mM β-mercaptoethanol, 0.1% NP-40) and incubated at 4°C with gentle rotation for at least 18 h. Magnetic Ni-NTA resin was washed once with 800 µL wash buffer 1 and three times with 800 µL PNK buffer (50 mM Tris-HCl (pH 7.8), 10 mM MgCl, 0.1% NP-40, 5mM β-mercaptoethanol). The magnetic resin was resuspended in 80 µL NP-PNK buffer (50 mM Tris-HCl (pH 7.8), 10 mM MgCl, 5mM β-mercaptoethanol) containing 8 U of thermosensitive alkaline phosphatase (TSAP, Promega) and 80 U of RNasin (Promega) and incubated at 20°C with rotation for 1 h. Ni-NTA resin was washed once with 800 µL wash buffer 1 and three times with 800 µL PNK buffer. The magnetic Ni-NTA resin was resuspended in 80 µL NP-PNK buffer containing 20 U of T4 polynucleotide kinase (NEB), 20 U of RNasin (Promega) and 30 µCi ^{32}P-ATP and incubated at 22°C with rotation for 100 min. Cold 1 mM ATP was spiked in and reaction allowed to proceed for another 50 min. Ni-NTA resin was again washed twice with 800 µL wash buffer 1 and four times with 800 µL PNK buffer and then resuspended in 80 µL of NP-PNK buffer containing 40 U of T4 RNA ligase I (NEB), 1 mM ATP, 20 U of RNasin (Promega) and 100 pM of an L5 index barcoded 5' linker (Integrated DNA Technologies). The ligation reaction was incubated at 16°C with gentle rotation for at least 12 h. The magnetic Ni-NTA resin was washed twice with 800 µL wash buffer 1 and four times with 800 µL PNK buffer, and resuspended in 60 µL NP-PNK buffer containing 40 U of T4 RNA ligase I (NEB), 20 U of RNasin (Promega) and 80 pM of 3' linker App-PE (Integrated DNA Technologies). The ligation reaction was incubated at 18°C with gentle rotation for 6 h and then Ni-NTA resin was washed twice with 800 µL wash buffer 1 and four times with 800 µL wash buffer 2 (50 mM Tris-HCl (pH 7.8), 50 mM NaCl, 0.1% NP-40, 5mM β-mercaptoethanol, 10 mM imidazole). ^{32}P-labelled RNase III.RNA

complexes were eluted in 60 μL of 1x NuPAGE LDS buffer (Thermo) and incubated at 65°C for 10 min with gentle shaking. Eluates were magnetically separated and loaded onto a NuPAGE 4-12% gradient Bis-Tris PAGE gel (Invitrogen) and run in 1x NuPAGE running buffer for ~1 h at 125 V. ^{32}P-labelled RNase III.RNA complexes were visualised by autoradiography, gel excised and recovered by incubating the fragmented gel pieces in 600 μL of wash buffer 3 (50 mM Tris-HCl (pH 7.8), 50 mM NaCl, 0.1% NP-40, 5mM β-mercaptoethanol, 1% SDS, 5 mM EDTA) and 100 μg of proteinase K at 55°C for 2 h with shaking. RNA was extracted using 3 M NaOAc and phenol:chloroform:isoamylalcohol, ethanol precipitated and pellet resuspended in 13 μL of RT buffer (RT_PE_reverse oligonucleotide, 5 mM dNTPs, H2O) and reverse-transcribed using Superscript III according to manufacturer's instructions. cDNA was incubated with RNase H (Thermo) at 37°C for 1 h and amplified using Phusion Hot Start Flex DNA Polymerase (NEB), 10 μM P5 and BC1 oligonucleotides. The cDNAs were amplified for 24 cycles with 2 μL of cDNA used as template. Products were separated on a 1.5% metaphor agarose gel and amplicons excised and purified using the MiniElute gel extraction kit (Qiagen). Libraries were pooled to equimolar, quantified on an Agilent bioanalyzer and underwent SPRI bead clean-up. The libraries were then quantified on an Agilent bioanalyzer for a second time and sequenced on an Illumina NextSeq500 platform with the addition of 2% PhiX DNA at Ramaciotti Centre for Genomics (University of New South Wales, Sydney), generating 150-cycle paired-end reads.

Analysis of RNase III-CLASH. Analysis of the CLASH data was similar to those described in Waters et al. (2017) and Tree *et al.* (2018). A Snakemake (Köster and Rahmann, 2012) pipeline was developed to automate all steps for the analysis of sequencing data for each CLASH experiment. The steps implemented in the Snakemake pipeline are described below and the scripts available in the GitHub repository (https://github.com/IgnatiusPang/Hyb-CRAC-R).

Merging paired-end reads and demultiplexing. The two halves of the paired-end reads were merged together using 'bbmerge' (Bushnell, 2014) with the following linker sequence (NAGATCGGAAGAGCACACGTCTG). Both the merged and unmerged reads were collated for demultiplexing. Demultiplexing was performed using the 'pyBarcodeFilter.py' script from 'pyCRAC' (Webb et al., 2014), this resulted in one FASTQ format file per sample barcode. The following analysis steps were performed on each demultiplexed FASTQ file, with one FASTQ file per sample.

Adapter and quality trimming. The 'bbduk' tool (Bushnell, 2014) was used to trim the sequences. The 3'-adapter sequences were right-trimmed using the option 'ktrim=r', where all sequences on the right-hand side of the sequences are removed from subsequent analyses. These 3'-adapter sequences include the following: adapter sequence (NAGATCGGAAGAGCACACGTCTG), Illumina universal adapter (AATGATACGGCGACCACCGAGATCTACACTCTTTCCCTACACGACGCTCTTCCGATCT), indexed adapter (AGATCGGAAGAGCACACGTCTGAACTCCAGTCACATC), PhiX adapters (AGATCGGAAGAGCGGTTCAGCAGGAATGCCGAGACCGATCTCGTATGCCGTCTT

CTGCTTGAAA and AGATCGGAAGAGCGTCGTGTAGGGAAAGAGTGTAGATCTCGGTGGTCGCCGTATCATTAAAAAA), the PhiX genome (NC_001422.1), and any poly A or poly T sequences. To specify the size of the kmers, the parameter 'k=3' specifies the kmer size to use for the majority of the length of the sequence and the 'mink=11' parameter specifies a shorter kmer size of 11 at the last 11 bases of the reads. At most one mismatch was allowed per sequence, this is specified with the 'hdist=1' and 'hdist2=1' parameters. For length trimming, the 'minlen=20' parameter would discard reads less than 20 nucleotides long after trimming. For quality trimming, the 'qtrim=rl' parameter specified trimming on both the right and left side of the sequence and the 'trimq=28' parameter will perform quality trimming to Q28 using the Phred algorithm. The "ordered" parameter keeps the reads in the same order as the input file when multiple CPU cores were used. To remove sequences that are of low complexity, the parameters 'entropy=0.2 entropywindow=20 entropyk=5' will filter out reads that have an average entropy of under 0.1 with a sliding window size of 20 and a kmer size of 5. A FASTQC (Andrews et al., 2012) analysis performed to enable visual inspection to ensure the trimmed output sequences are of high quality.

Removal of PCR duplicates, and detection and annotation of hybrids. Reads as a result of PCR duplicates were removed using the pyFastqDuplicateRemover.py script from the pyCRAC library (Webb et al., 2014). The 'hyb detect' function of the hyb pipeline (Travis et al., 2014) was used to detect the genomic co-ordinates of the two RNA sequences from the linked sequence hybrid of the RNA-RNA interaction. Multiple reads for the same RNA-RNA interaction were counted and the sequences merged using the 'hyb merge' function of the hyb pipeline. This resulted in a table of RNA-RNA interactions with a count for the number of reads supporting each RNA-RNA interaction. Only hybrid sequences in which the two RNA sequences were unambiguously mapped to two corresponding genomic locations were used for further analysis. Hybrid sequences in which two RNA sequences were less than 100 or equal to nucleotides apart from each other were excluded from further analyses. Each RNA transcript from the RNA-RNA interaction were annotated by comparing the genomic co-ordinates with the transcriptome annotations from ANNOgesic pipeline. Annotations were assigned based on priority, ranked from highest to lowest priority. These priorities are ncRNA, CDS, 5' UTR, 3' UTR, rRNA, tRNA, CRISPR, pseudogene, riboswitch, Ribonuclease P RNA, RNA thermometer, Signal recognition particle RNA, Transfer-messenger RNA, and operon.

Obtaining read counts of RNA transcripts. The read counts of individual RNA transcripts corresponding to those identified in the hybrid sequences was obtained from the merged and trimmed paired-end reads data using the 'CRAC_pipeline_SE.py' script from the 'pyCRAC' pipeline. The sequence alignment within the 'pyCRAC' pipeline was performed using 'NovoAlign' (http://www.novocraft.com/products/novoalign/). For each of the two RNA sequences from a hybrid sequence, the read count corresponds to the highest read depth within the region spanned by the RNA sequence. The output from the 'pyCRAC' pipeline is in SGR format which was converted into PWS format using a custom python script (sgr2pws.py).

Probabilistic analysis of RNA-RNA interactions. In order to identify RNA-RNA interactions that are of biological origin, we compared each observed interaction with the

background probability that the same RNA-RNA interaction event could occur randomly *in vitro*. The analysis was based on the method described in Sharma *et al.* (2016) and Waters *et al.* (2017). Briefly, the probability of drawing a gene at random, $P(g_x)$, was estimated by the maximum read depth for the gene divided by the total number of reads in the replicate (N). The background probability for observing a RNA-RNA interaction, pdf (gx, gy), was estimated by multiplying the probabilities $P(g_x)$ and $P(g_y)$. Ligation events that were not observed were set to a probability of zero, as per Sharma *et al.* (2016). The probability distribution $pdf(g_x, g_y)$ was re-normalized to the sum of 1. The background probability of observing k occurrences of interaction between gx and gy was modeled using the binomial distribution, $k \sim Binomial(p=pdf(g_x, g_y), N)$. These calculations were used to assign a p-value (ie: $P(X=k)$) to each experimentally observed pair of RNA-RNA interaction. For RNA-RNA interactions that were observed in multiple biological replicate experiments, the corrected p-value was combined using the Fisher's method. If the RNA-RNA interaction was only identified in one biological replicate, the original p-value was used and Fisher's method was not applied. After processing with Fisher's method, the p-values were corrected for false discovery rates using the Benjamini-Hochberg multiple testing correction. Statistically significant RNA-RNA interactions were defined as those with a false discovery rate threshold of $p\text{-value} < 0.05$ (Tree et al., 2018).

References

Andrews, S., Krueger, F., Segonds-Pichon, A., Biggins, L., Krueger, C., Wingett, S., 2012. FastQC. Babraham, UK.

Bushnell, B., 2014. BBMap: A Fast, Accurate, Splice-Aware Aligner.

Dar, D., Shamir, M., Mellin, J.R., Koutero, M., Stern-Ginossar, N., Cossart, P., Sorek, R., 2016. Term-seq reveals abundant ribo-regulation of antibiotics resistance in bacteria. Science 352, aad9822. https://doi.org/10.1126/science.aad9822

Dugar, G., Herbig, A., Förstner, K.U., Heidrich, N., Reinhardt, R., Nieselt, K., Sharma, C.M., 2013. High-resolution transcriptome maps reveal strain-specific regulatory features of multiple Campylobacter jejuni isolates. PLoS Genet. 9, e1003495. https://doi.org/10.1371/journal.pgen.1003495

Köster, J., Rahmann, S., 2012. Snakemake—a scalable bioinformatics workflow engine. Bioinformatics 28, 2520–2522. https://doi.org/10.1093/bioinformatics/bts480

Li, H., Durbin, R., 2009. Fast and accurate short read alignment with Burrows-Wheeler transform. Bioinforma. Oxf. Engl. 25, 1754–1760. https://doi.org/10.1093/bioinformatics/btp324

Li, H., Handsaker, B., Wysoker, A., Fennell, T., Ruan, J., Homer, N., Marth, G., Abecasis, G., Durbin, R., Subgroup, 1000 Genome Project Data Processing, 2009. The Sequence Alignment/Map format and SAMtools. Bioinformatics 25, 2078–2079. https://doi.org/10.1093/bioinformatics/btp352

Quinlan, A.R., Hall, I.M., 2010. BEDTools: a flexible suite of utilities for comparing genomic features. Bioinformatics 26, 841–842. https://doi.org/10.1093/bioinformatics/btq033

Sharma, C.M., Vogel, J., 2014. Differential RNA-seq: the approach behind and the biological insight gained. Curr. Opin. Microbiol. 19, 97–105. https://doi.org/10.1016/j.mib.2014.06.010

Sharma, E., Sterne-Weiler, T., O'Hanlon, D., Blencowe, B.J., 2016. Global Mapping of Human RNA-RNA Interactions. Mol. Cell 62, 618–626.

https://doi.org/10.1016/j.molcel.2016.04.030
Travis, A.J., Moody, J., Helwak, A., Tollervey, D., Kudla, G., 2014. Hyb: A bioinformatics pipeline for the analysis of CLASH (crosslinking, ligation and sequencing of hybrids) data. Methods 65, 263–273. https://doi.org/10.1016/j.ymeth.2013.10.015
Tree, J.J., Gerdes, K., Tollervey, D., 2018. Transcriptome-Wide Analysis of Protein–RNA and RNA–RNA Interactions in Pathogenic Bacteria, in: Methods in Enzymology. Elsevier, pp. 467–488. https://doi.org/10.1016/bs.mie.2018.08.009
Waters, S.A., McAteer, S.P., Kudla, G., Pang, I., Deshpande, N.P., Amos, T.G., Leong, K.W., Wilkins, M.R., Strugnell, R., Gally, D.L., Tollervey, D., Tree, J.J., 2017. Small RNA interactome of pathogenic *E. coli* revealed through crosslinking of RN ase E. EMBO J. 36, 374–387. https://doi.org/10.15252/embj.201694639
Webb, S., Hector, R.D., Kudla, G., Granneman, S., 2014. PAR-CLIP data indicate that Nrd1-Nab3-dependent transcription termination regulates expression of hundreds of protein coding genes in yeast. Genome Biol. 15, R8. https://doi.org/10.1186/gb-2014-15-1-r8
Weber, C.M., Ramachandran, S., Henikoff, S., 2014. Nucleosomes are context-specific, H2A.Z-modulated barriers to RNA polymerase. Mol. Cell 53, 819–830. https://doi.org/10.1016/j.molcel.2014.02.014
Yu, S.-H., Vogel, J., Förstner, K.U., 2018. ANNOgesic: a Swiss army knife for the RNA-seq based annotation of bacterial/archaeal genomes. GigaScience 7. https://doi.org/10.1093/gigascience/giy096
