# Supplementary material for: RNase III-CLASH of multi-drug resistant Staphylococcus aureus reveals a regulatory mRNA 3′UTR required for intermediate vancomycin resistance
Source: Nat Commun. 2022 Jun 22;13:3558. doi: 10.1038/s41467-022-31177-8 (PMC9217812; doi:10.1038/s41467-022-31177-8)
Supplement: Supplementary file 3 — Description of Additional Supporting Information [file 41467_2022_31177_MOESM3_ESM.pdf]

**RNase III-CLASH of multi-drug resistant *Staphylococcus aureus* reveals a regulatory mRNA 3'UTR required for intermediate vancomycin resistance**

Daniel G. Mediati, Julia L. Wong, Wei Gao, Stuart McKellar, Chi Nam Ignatius Pang, Sylvania Wu, Winton Wu, Brandon Sy, Ian R. Monk, Joanna M. Biazik, Marc R. Wilkins, Benjamin P. Howden, Timothy P. Stinear, Sander Granneman, Jai J. Tree

## DESCRIPTION OF ADDITIONAL SUPPLEMENTARY DATA

**Title:** Supplementary Data 1

Transcription start sites and RNA 3' ends determined by dRNA-seq and Term-seq analysis in *Staphylococcus aureus* strain JKD6009 (Genbank: LR027876.1).

- Tab 1 provides a description of the data and header for data presented in Tab2
- Tab 2 provides start and end positions detected in the JKD6009 transcriptome
- Tab 3 provides the same data as Tab 2 in GFF format.

**Title:** Supplementary Data 2

List of known and predicted sRNA from ANNOgesic analysis of RNA-seq, dRNA-seq, and Term-seq data generated from *Staphylococcus aureus* strain JKD6009 (Genbank: LR027876.1).

- Tab 1 provides a description of the data and header for data presented in Tab2
- Tab 2 provides all small RNA previously reported and predicted by ANNOgesic in the JKD6009 transcriptome
- Tab 3 provides the same data as Tab 2 in GFF format.

**Title:** Supplementary Data 3

RNA-RNA interactions recovered by RNase III-CLASH. All hybrid reads representing statistically significant RNA-RNA interactions. Data is collated from all RNase III-CLASH experiments reported here and in the parallel study by McKellar *et al* Nature Communications 2022.

- Tab 1 provides all statistically significant RNA-RNA interactions represented by all hybrid reads recovered by RNase III-CLASH. NB: clusters of hybrid reads that were recovered in independent experiments are reported as "Merged" and details of these read clusters are provided in Tab3.
- Tab 2 provides a description of the data and header for data presented in Tab1
- Tab 3 provides detailed information about clusters of hybrid reads that were recovered in multiple independent experiments.

**Title:** Supplementary Data 4

Strains, Plasmids, and Oligonucleotides used in this study.

- Tab 1 provides information about bacterial strains used in this study
- Tab 2 provides information about plasmids used in this study
- Tab 3 provides information about oligonucleotides used in this study
